# Phylogenomic Analysis Reveals an Asian Origin for African *Burkholderia pseudomallei* and Further Supports Melioidosis Endemicity in Africa

**DOI:** 10.1128/mSphere.00089-15

**Published:** 2016-03-09

**Authors:** Derek S. Sarovich, Benoit Garin, Birgit De Smet, Mirjam Kaestli, Mark Mayo, Peter Vandamme, Jan Jacobs, Palpouguini Lompo, Marc C. Tahita, Halidou Tinto, Innocente Djaomalaza, Bart J. Currie, Erin P. Price

**Affiliations:** a Global and Tropical Health Division, Menzies School of Health Research, Darwin, Australia; b Bacteriological Unit, Institut Pasteur de Madagascar, Antananarivo, Madagascar; c Department of Clinical Sciences, Institute of Tropical Medicine, Antwerp, Belgium; d Faculty of Sciences, Laboratory of Microbiology, Ghent University, Ghent, Belgium; e Department of Microbiology and Immunology, University of Leuven, Leuven, Belgium; f Clinical Research Unit of Nanoro (IRSS-CRUN), Nanoro, Burkina Faso; g Androva University Hospital, Mahajanga, Madagascar; University of Wisconsin—Madison

**Keywords:** *Burkholderia*, epidemiology, infectious disease, melioidosis, phylogeography, population genetics

## Abstract

*Burkholderia pseudomallei*, an environmental bacterium that causes the deadly disease melioidosis, is endemic in northern Australia and Southeast Asia. An increasing number of melioidosis cases are being reported in other tropical regions, including Africa and the Indian Ocean islands. *B. pseudomallei* first emerged in Australia, with subsequent rare dissemination event(s) to Southeast Asia; however, its dispersal to other regions is not yet well understood. We used large-scale comparative genomics to investigate the origins of three *B. pseudomallei* isolates from Madagascar and two from Burkina Faso. Phylogenomic reconstruction demonstrates that these African *B. pseudomallei* isolates group into a single novel clade that resides within the more ancestral Asian clade. Intriguingly, South American strains reside within the African clade, suggesting more recent dissemination from West Africa to the Americas. Anthropogenic factors likely assisted in *B. pseudomallei* dissemination to Africa, possibly during migration of the Austronesian peoples from Indonesian Borneo to Madagascar ~2,000 years ago, with subsequent genetic diversity driven by mutation and recombination. Our study provides new insights into global patterns of *B. pseudomallei* dissemination and adds to the growing body of evidence of melioidosis endemicity in Africa. Our findings have important implications for melioidosis diagnosis and management in Africa.

**IMPORTANCE** Sporadic melioidosis cases have been reported in the African mainland and Indian Ocean islands, but until recently, these regions were not considered areas where *B. pseudomallei* is endemic. Given the high mortality rate of melioidosis, it is crucial that this disease be recognized and suspected in all regions of endemicity. Previous work has shown that *B. pseudomallei* originated in Australia, with subsequent introduction into Asia; however, the precise origin of *B. pseudomallei* in other tropical regions remains poorly understood. Using whole-genome sequencing, we characterized *B. pseudomallei* isolates from Madagascar and Burkina Faso. Next, we compared these strains to a global collection of *B. pseudomallei* isolates to identify their evolutionary origins. We found that African *B. pseudomallei* strains likely originated from Asia and were closely related to South American strains, reflecting a relatively recent shared evolutionary history. We also identified substantial genetic diversity among African strains, suggesting long-term *B. pseudomallei* endemicity in this region.

## Abstract (precis)

Sporadic melioidosis cases have been reported in the African mainland and Indian Ocean islands, but until recently, these regions were not considered areas where *B. pseudomallei* is endemic. Given the high mortality rate of melioidosis, it is crucial that this disease be recognized and suspected in all regions of endemicity. Previous work has shown that *B. pseudomallei* originated in Australia, with subsequent introduction into Asia; however, the precise origin of *B. pseudomallei* in other tropical regions remains poorly understood. Using whole-genome sequencing, we characterized *B. pseudomallei* isolates from Madagascar and Burkina Faso. Next, we compared these strains to a global collection of *B. pseudomallei* isolates to identify their evolutionary origins. We found that African *B. pseudomallei* strains likely originated from Asia and were closely related to South American strains, reflecting a relatively recent shared evolutionary history. We also identified substantial genetic diversity among African strains, suggesting long-term *B. pseudomallei* endemicity in this region.

## INTRODUCTION

The soil-dwelling bacterium *Burkholderia pseudomallei* is the etiological agent of melioidosis, a disease with high mortality rates in humans and many animals (1). Melioidosis has diverse clinical manifestations and can be difficult to diagnose microbiologically, even in technologically sophisticated laboratories (2, 3). Because of the high mortality rate of melioidosis, which ranges from 10 to 40% even with treatment, the intrinsic resistance of *B. pseudomallei* to many antibiotics, the potential for acquisition via the aerosol route, and the lack of an effective vaccine, this bacterium is among a small group of pathogens listed as Tier 1 Select Agents by the United States government (1, 4).

The true global distribution of *B. pseudomallei* remains unclear, although regions not previously thought to harbor this organism are now being identified (1, 5–7). Outside the known hyperendemicity foci of northern Australia and Southeast Asia, *B. pseudomallei* has been sporadically found in most other tropical regions, including Africa, the Indian Ocean islands, and the Americas (8). It remains unclear whether the spread of melioidosis beyond the known regions where *B. pseudomallei* is endemic has occurred in recent decades or if it is just being unmasked in Africa and the Americas by better recognition and increased surveillance (9). Several African mainland countries have reported suspected locally acquired melioidosis cases, including Gabon, Malawi, and Sierra Leone (10–12), and there are several instances of travelers acquiring melioidosis during visits to Africa (13–15). Cases have also been reported in Madagascar and the Indian Ocean islands of Mauritius, Seychelles, and La Réunion (16–19). Additionally, countries in Central and South America, including Brazil, Colombia, Ecuador, and Honduras, and several islands in the Caribbean, including Guadeloupe, Martinique, and Puerto Rico, have also reported melioidosis cases (20–31), suggesting that *B. pseudomallei* is endemic in almost all tropical regions across the globe, as recently predicted by an extensive global modeling study (32).

Although *B. pseudomallei* has one of the most highly recombinogenic genomes identified to date (33), phylogeographic reconstruction and subsequent geographic attribution of *B. pseudomallei* populations is possible. The strong geographic signal present in the *B. pseudomallei* genome reflects its primary mode of transmission; melioidosis is almost invariably caused by contact with contaminated surface water or soil, with human-to-human transmission being exceptionally rare. Thus, large geographic barriers (e.g., the Wallace Line, which separates most of Asia from Australasia) have substantially restricted gene flow between populations. These factors have led to distinct Asian and Australasian *B. pseudomallei* populations that have regionally evolved over hundreds or thousands of years (33, 34).

Multilocus sequence typing (MLST) is commonly used to study the population structure of many pathogens, including *B. pseudomallei* (http://pubmlst.org/bpseudomallei), and can be a useful tool for determining the geographic origin of *B. pseudomallei* strains (34, 35). More recently, an inexpensive capillary electrophoresis-based typing method that exploits size and allelic variations in the 16S-23S rDNA intergenic transcribed spacer (ITS) region has been used to detect geographic signal and diversity in *B. pseudomallei* (36, 37). However, we and others have noted that these typing methods are unable to determine fine-scale population structure because of low genotyping resolution and high rates of recombination among local *B. pseudomallei* populations (33, 35). In addition, we have shown that MLST can, albeit infrequently, lead to confounding results because of sequence type (ST) homoplasy (38). Thus, phylogenetic reconstruction of *B. pseudomallei* populations using genome-wide single-nucleotide polymorphisms (SNPs) derived from whole-genome data is needed to identify the true geographic origins of strains.

In 2009, using emerging next-generation whole-genome sequencing (WGS) technologies, Pearson and coworkers were the first to hypothesize that *B. pseudomallei* originated in Australia, followed by one or more rare transmission events to Southeast Asia. Using Bayesian inference and molecular clock estimates, Pearson et al. estimated that *B. pseudomallei* moved into Southeast Asia during the last glacial period (between 16 and 225 thousand years ago), when the Sahul and Sunda land masses were in closest proximity because of greatly lowered sea levels. Subsequently, *B. pseudomallei* has disseminated to other parts of the globe via similarly rare but significant transmission events (33). This seminal study by Pearson and coworkers was based on relatively few strains, but subsequent studies across larger and more diverse *B. pseudomallei* data sets have supported their initial hypothesis (35, 38, 39). Although the relationship between Asian and Australian strains has been reasonably well characterized, the origin of *B. pseudomallei* in other parts of the world is still poorly understood. Of particular interest are Africa and the Americas, where melioidosis was once considered sporadic but is now being unmasked as likely endemic (5). In this study, we aimed to further characterize the global phylogeography of *B. pseudomallei* by using large-scale comparative genomics to determine the origin of melioidosis in Africa, which has not yet been investigated on a whole-genome scale.

## RESULTS AND DISCUSSION

### Phylogenomic analysis identifies an African and South American *B. pseudomallei* clade.

**FIG 1:**
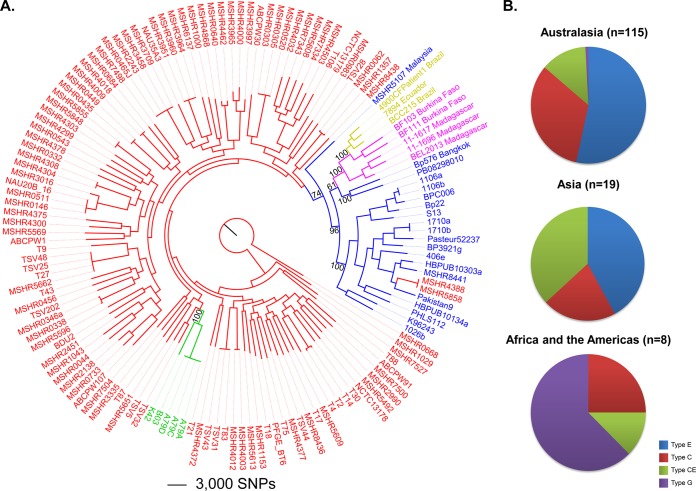
Genetic and genomic comparisons of the African *B. pseudomallei* isolates with global *B. pseudomallei* reference strains. (A) Maximum-parsimony phylogeny of 144 *B. pseudomallei* genomes. This tree was drawn by using 202,245 core genome, orthologous, bi-allelic SNPs identified by the SPANDx pipeline (75) and rooted by using MSHR0668. Isolates are color coded by origin as follows: Australia, red; Papua New Guinea, green; Asia, blue; Africa (including Madagascar), pink; South America, gold. African and South American isolates form their own clade that falls within the more ancestral Asian clade, which is separate from Australasian *B. pseudomallei*. Bootstrap values were generated on the basis of 200 replicates. The consistency index is 0.19. (B) Distribution of ITS types by geographic region. Similarly to earlier studies, isolates from Africa and the Americas show an overrepresentation of ITS type G in comparison to strains from Australasia and Asia; however, African isolates also possess ITS types CE and C, alleles that are commonly identified in isolates from regions of Asia and Australasia where *B. pseudomallei* is hyperendemic.

Comparative analysis of the 144 *B. pseudomallei* genomes, including the five new African genomes generated in this study, identified 202,245 bi-allelic SNPs among these strains. These SNPs were used to reconstruct a maximum-parsimony phylogenetic tree (Fig. 1A). As previously shown (33, 35, 38, 39), we observed a clear distinction between Australasian (red or green) and Asian (blue) *B. pseudomallei* strains, which was supported by a bootstrap value of 74%. The separation of the Australasian and Asian *B. pseudomallei* clades is due to the restricted dispersal of *B. pseudomallei* between these two geographic regions, with ST-562 being the only example where a recent overlap between these two regions has been confirmed (39, 40). The African (pink) and South American (gold) strains all reside on a separate, highly supported (bootstrap value = 100%) branch within the Asian clade.

Interestingly, the three South American strains (4900CFPatient1 [36, 41], 7894 [42], and BCC215 [24]) share a node with the two Burkina Faso strains, although the Burkina Faso strains are more closely related to each other than to the South American strains; in contrast, the three Madagascan isolates reside on a separate branch within the African-South American clade (bootstrap value = 100%). This observation demonstrates that African and South American *B. pseudomallei* likely originated from an Asian ancestor, with no evidence of *B. pseudomallei* transmission directly from Australia. The location of the African and South American strains within the Asian clade also confirms that transmission into Africa must have occurred after the first *B. pseudomallei* transmission from Australia to Asia, which has been estimated to have occurred between 16,000 and 225,000 years ago (33). Further, our analysis hints at African ancestry for South American strains, with these strains emerging from a relatively recent branch within the deeper African node (Fig. 1A). This finding suggests that *B. pseudomallei* was transmitted from West Africa to the Americas. Genomic data from additional American *B. pseudomallei* isolates will provide much-needed insight into this possibility.

We were next interested in investigating the region in Asia where African *B. pseudomallei* originated. The Asian isolates closest to the African and South American strains in our phylogeny are *B. pseudomallei* 576 and PB08298010. However, this branch has only moderate bootstrap support (61%), likely because of undersampling of this clade in the current data set, so we were unable to identify the precise origin of this ancestor. *B. pseudomallei* 576 was obtained from a 49-year-old female from Bangkok, Thailand, and *B. pseudomallei* PB08298010 was isolated from a melioidosis case that occurred in Arizona, United States, and was speculated to have originated from contaminated medical supplies imported from Malaysia (35). In support of an Asian origin for PB08298010, its MLST profile is ST-426, which matches the ST of MSHR0158, an isolate from an Australian traveler thought to have acquired melioidosis in Penang, Peninsular Malaysia (http://pubmlst.org/bpseudomallei). Our global phylogeny also supports an Asian origin for *B. pseudomallei* PB08298010, although this strain does not group with a second Malaysian isolate included in our analysis (MSHR5107). However, unlike MSHR0158, MSHR5107 was isolated from Sarawak, Malaysian Borneo. ST data show little overlap between the Peninsular Malaysia and Malaysian Borneo isolates; thus, it is not surprising that PB08298010 and MSHR5107 do not group together on a whole-genome level.

Our phylogeny demonstrates support for African strains being of Asian origin, although a greater number of Asian strains from more diverse locations will enable more accurate identification of the precise origin(s) of African *B. pseudomallei*. In particular, there are several suspected regions of endemicity in Asia that have no representative WGS data in our phylogeny, such as Indonesia, Philippines, and Myanmar. Additional isolates from these locations will increase the resolution of these poorly populated clades and may potentially identify a more precise origin of African *B. pseudomallei*. Of note, genetic analysis of the Malagasy, the indigenous people of Madagascar, shows that approximately half have shared ancestry with Indonesian Borneo people (43). Austronesian migration from Indonesian Borneo to Madagascar is thought to have occurred between 1,500 and 2,000 years ago (44, 45). This migration provides a possible date for an anthropogenically driven introduction of Asian *B. pseudomallei* into Madagascar and subsequent transfer from Madagascar to the African mainland, potentially through human migration, trade, or slavery routes. At the time of this Austronesian migration, pigs were also introduced to Madagascar (46). Pigs are known to become infected with *B. pseudomallei*, although they are generally less susceptible than sheep, goats, or horses (47, 48); it is plausible that pigs acted as a reservoir for this pathogen, facilitating the transfer of *B. pseudomallei* into Madagascar. Several studies have shown that certain bird species may be infected by or commensally carry *B. pseudomallei* (47, 49). Therefore, an additional possibility is that *B. pseudomallei* was disseminated from Asia to Madagascar by migratory birds flying along the Asia-East Africa flyway, similar to what has been observed in the introduction of West Nile virus to the Americas from West Africa (50). Future phylogenomic and molecular clock investigations using a larger number of strains from Southeast Asia, Africa, and the Americas will provide further important clues to the origin of *B. pseudomallei* in Africa and the Americas.

### MLST hints at deeper phylogenetic relationships but is subject to homoplasy.

The three clinical isolates from Madagascar belong to STs 1043, 1053, and 1054 (Table 1; see Table S3 in the supplemental material). STs 1043 and 1053 are single-locus variants, whereas ST-1054 is a double-locus variant of ST-1053 and a triple-locus variant of ST-1043. All three of these STs share identical *ace*, *lipA*, *narK*, and *ndh* alleles but differ at the remaining three loci (*gltB*, *gmhD*, and *lepA*). The two isolates from Burkina Faso belong to STs 1122 and 1121 and are triple-locus variants of one another. STs 1122 and 1043 are double-locus variants, although one strain originated in Madagascar and the other originated in Burkina Faso, a relationship that is potentially misleading and not reflected on a whole-genome scale (Fig. 1A). All five STs are unique, with no other matches in the *B. pseudomallei* MLST database, including other characterized strains from Africa and South America (see Table S3 in the supplemental material). Although MLST shows some utility for determining strain origin, it can be unreliable in highly recombinogenic organisms like *B. pseudomallei* when trying to determine deeper phylogenetic relationships (51). Such homoplasy has been demonstrated previously, where *B. pseudomallei* isolates from Cambodia and Australia were identical by MLST but differed greatly on a whole-genome level (38), thereby making accurate geographic attribution in these cases impossible without WGS. One conclusion that can be made is that there is considerable underlying genetic diversity in these regions, a finding supported by our WGS phylogeny (Fig. 1A) and noted previously in multiple studies using less highly resolving typing methods (13, 16). This underlying diversity further supports the notion that the introduction of *B. pseudomallei* to these regions is not a recent event, as sufficient time has elapsed since their introduction for divergence to occur.

**TABLE 1:** African and American *B. pseudomallei* isolates used in this study and associated genotyping information

Strain name	Other name(s)	ST^b^ **(allelic profile)**	ITS type	Geographic origin, reference (GenBank accession no.)
11-1617	MSHR7969	1054 (4, 12, 34, 1, 5, 2, 1)	CE	Madagascar, 16 (SRR3145392)
11-1696	MSHR7968	1053 (4, 12, 3, 2, 5, 2, 1)	C	Madagascar, 16 (SRR3145393)
BEL2013	MSHR7966	1043 (4, 1, 3, 2, 5, 2, 1)	G	Madagascar, 62 (SRR3145396)
BF103	ITM BF103, MSHR7964	1121 (1, 18, 13, 2, 5, 6, 1)	G	Burkina Faso (SRR3145394)
BF111	ITM BF111, MSHR7965	1122 (1, 1, 19, 2, 5, 2, 1)	C	Burkina Faso (SRR3145395)
4900CFPatient1	NA^a^	92 (1, 1, 2, 1, 5, 1, 1)	G	Brazil (ARZE00000000)
7894	7894/300	11 (1, 1, 13, 1, 6, 1, 1)	G	Ecuador (CP009535, CP009536)
BCC215	NA	1355 (1, 156, 2, 2, 5, 1, 1)	G	Ceará, Brazil (ABBR00000000)

a NA, not applicable.

b Per the *B. pseudomallei* MLST database (http://pubmlst.org/bpseudomallei).

### ITS typing shows unexpected diversity in African, but not American, isolates.

Previously, ITS typing of 1,191 *B. pseudomallei* isolates revealed that strains from Africa and the Americas have restricted diversity at the 16S-23S rDNA ITS locus, with a dominance of the ITS type G allele among these isolates (36, 37). However, prior to this study, only four African strains had been examined by ITS typing (2002721628 from Madagascar [ITS type G], 2002721629 and 2002721639 from Kenya [ITS types G and C, respectively], and 2002721691 from Mauritius [ITS type GC]) (37). Using WGS data, we determined the ITS genotypes of all of the strains in our data set, including the five African strains. Australasian isolates (*n =* 115; MSHR6137 could not be typed because of a lack of sequence information covering the 16S-23S spacer region) were predominantly ITS type E (53%), followed by ITS type C (33%) and ITS type CE (13%), with a very small percentage of ITS type G isolates (<1%). Asian isolates (*n =* 19; excluding PB08298010 due to uncertain provenance) were mostly ITS type CE (42%), followed by ITS type E (37%) and ITS type C (21%). The distribution of Australasian ITS types was almost identical to that seen previously (37); however, the distribution of ITS types among Asian strains differed somewhat, most likely because of sampling bias incurred by our small data set. We did not identify an ITS type G isolate among our Asian strains, which was expected, given the rarity of type G in Asia (<1%) (37). Although the precise origin of PB08298010 is not known, it has previously been shown to be ITS type G (36). The three Madagascan isolates were ITS types C, CE, and G, and the two Burkina Faso isolates were ITS types C and G (Table 1; Fig. 1B). These results reveal further diversity among *B. pseudomallei* strains from Africa, with the CE allele not previously reported.

The finding of an additional ITS type in the African isolates indicates a complex evolutionary history of *B. pseudomallei* in this region that is not yet well understood. We pose three possible explanations for this diversity. First, the introduction of *B. pseudomallei* to Africa from Asia may have occurred as a single transmission event that involved a mixed population of strains, and thus ITS types, with this ITS diversity persisting to the present day. Second, multiple introduction events with different strains may have occurred following several independent dissemination events from Asia. Third, the ITS region in some strains may have undergone convergent evolution across distinct geographic regions, perhaps because of similar selective pressures on *B. pseudomallei* populations. The ostensible rarity of ITS type G in Asia suggests that the introduction of this ITS allele to Africa was a highly improbable event, although comprehensive analysis of ITS allele distribution across Asian isolates, particularly those from Indonesian Borneo, is currently lacking. To date, ITS type G has been identified in only three Thai strains (Songkhla 25 W-1, Songkhla 25 W-2, and Phattalung 52 W-1). This observation is consistent with the node shared by African strains and Bangkok *B. pseudomallei* 576 on the whole-genome phylogeny, and it is therefore possible that ITS type G *B. pseudomallei* in Africa originated from Thailand. All 24 Malaysian strains examined in the study of Liguori et al. (37) lacked the ITS type G allele, although these isolates were derived from a historic *B. pseudomallei* collection and it is therefore unclear what proportion of these strains, if any, are from Malaysian Borneo. In addition, the heavy sampling bias toward isolates from Thailand (443/480 [92%] Asian strains) may explain the low abundance of ITS type G reported to date. Further ITS typing and WGS data from Bornean and African strains are needed to explore these hypotheses, with such data likely to assist in the more precise determination of the Asian ancestor of ITS type G in Africa.

In contrast to the African strains, the 18 American strains characterized by the ITS typing method have, to date, been genotyped only as ITS type G (36), suggesting a genetic bottleneck during *B. pseudomallei* dissemination from Africa to the Americas. This finding is consistent with our genome phylogeny, which hints at recent transmission of *B. pseudomallei* from Africa to the Americas. Although the route of this transmission is highly speculative, one possibility is the movement of West Africans to South America during the transatlantic slave trade between the 16th and 19th centuries. Using pulsed-field gel electrophoresis and MLST, a study of *B. pseudomallei* isolates from a 2003 Brazilian melioidosis outbreak showed some genetic diversity among strains (24), supporting our hypothesis that the introduction of *B. pseudomallei* to the Americas did not occur very recently. In addition, our WGS phylogeny confirmed that the three South American strains are not homogeneous on a whole-genome level (Fig. 1A). Whether this diversity also extends to the 16S-23S ITS region in other South American isolates remains to be elucidated.

### African *B. pseudomallei* strains vary in their virulence gene content.

**FIG 2:**
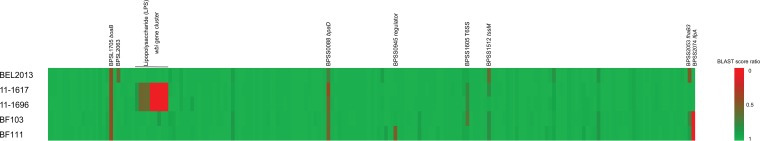
Blast score similarity ratios of all of the known virulence markers in *B. pseudomallei* K96243 compared to the African *B. pseudomallei* strains. African strains possess variable virulence gene contents, with trimeric autotransporter adhesions, LPS (*wbi* cluster), filamentous hemagglutinin (*fhaB3*), and lactonase family protein A (*lfpA*) all showing variability across strains.

*B. pseudomallei* has an impressive virulence arsenal, including capsular polysaccharide I, lipopolysaccharides (LPS), a diverse complement of autotransporters, multiple specialized secretion systems, a lethal toxin, and iron-scavenging siderophores (52). To assess the virulence potential of African *B. pseudomallei* strains, assembled genomes were screened against a large panel of known and putative *B. pseudomallei* virulence genes (Fig. 2; see Table S2 in the supplemental material). All African isolates and the two isolates from Brazil lacked *boaB* (*BPSL1705*), which encodes a trimeric autotransporter adhesin (TAA) that may have a role in macrophage invasion and survival (53); however, *boaB* was also absent from 81 other *B. pseudomallei* strains, including Australian clinical blood isolate MSHR0668 and Thai clinical liver abscess isolate 1106a (54), and is therefore unlikely to be a crucial virulence factor. Two additional TAAs were found to be variably present among the African strains; *bpaD* (*BPSS0088*) was absent from all five African strains, and *BPSL2063* (55) was absent from Madagascan strain BEL2013. Although *bpaD* was also variably present across the larger data set (absent from ~25% of the strains), *BPSL2063* was conserved across the other 142 genomes and variable in only one other isolate (*B. pseudomallei* Pakistan9; BLAST score ratio [BSR] = 0.72). The best characterized TAA, *Burkholderia* intracellular motility A (encoded by *bimA* [*BPSS1492*]), which is responsible for the actin-based motility of *B. pseudomallei* (56), was found in all of the African strains. These strains encoded for the more common *bimA*_Bp_ variant, which is associated with primary pneumonia presentation of melioidosis (57). The less common variant of *bimA*, *bimA*_Bm_, is found in ~12% of Australian strains. Outside Australia, it has been reported in only a single isolate from India (58); the *bimA*_Bm_ variant is strongly associated with neurological melioidosis (57).

**FIG 3:**
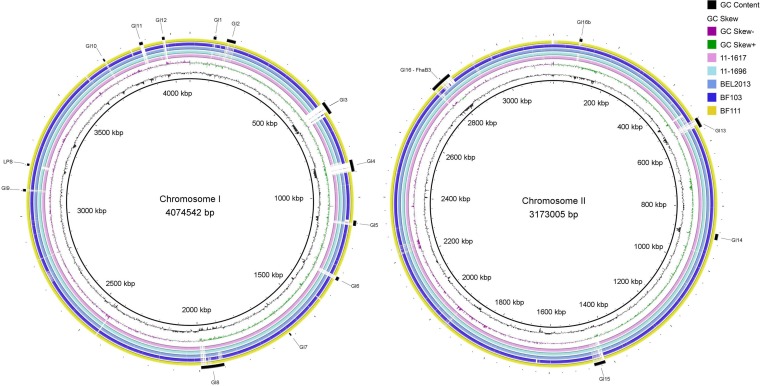
Whole-genome comparison of *B. pseudomallei* African strains aligned with *B. pseudomallei* K96243 by BLAST ring image generator. The outer ring shows the locations of the known genomic islands in the *B. pseudomallei* chromosomes. Also shown is the 13.5-kb deletion of the LPS locus in two of the Madagascan *B. pseudomallei* strains, 11-1617 and 11-1696, at positions ~3,198,200 to ~3,211,800 relative to strain K96243.

In addition to their variable TAA content, the African *B. pseudomallei* strains were highly varied at multiple genomic islands (GIs), including at GI16, which encodes two virulence factors, filamentous hemagglutinin (*fhaB3*) and lactonase family protein A (*lfpA*) (Fig. 2 and 3). Three out of five African *B. pseudomallei* strains contained the full-length *fhaB3* gene; however, BF103 (Burkina Faso) contained a truncated version (BSR = 0.77) and BEL2013 (Madagascar) lacked *fhaB3* entirely. The *fhaB3* gene is variably present in *B. pseudomallei* and was absent from or truncated in 24 additional strains across the larger data set. Absence of this gene has been previously associated with localized skin abscess formation without septicemia, a less severe form of melioidosis that is rarely fatal (57). An additional virulence factor located directly downstream of *fhaB3*, *lfpA*, is able to modify host macrophage expression to resemble osteoclastogenesis and is required for full virulence in the Syrian hamster model (59). The *lfpA* gene is absent from the two Burkina Faso strains but is present in all three strains from Madagascar. The presence of *lfpA* was highly variable across the remainder of the data set, being absent from ~50% of the strains.

The type II O-antigenic polysaccharide (O-PS; *wbi* gene cluster) is absent from two of the isolates from Madagascar (11-1617 and 11-1696). There is an ~14-kb deletion (genomic coordinates 3,198,200 to 3,211,800 relative to *B. pseudomallei* K96243 chromosome I), encompassing *rmlCD*, *wzm*, *wzt*, and *wbiABCDEFGHI* (Fig. 3). Type II O-PS has previously been shown to be required for serum resistance and virulence in animal models (60). Interestingly, this locus also shows a propensity for loss-of-function mutations in a persistent-carriage infection of an Australian patient who has been colonized with *B. pseudomallei* for >14 years (61). The type II O-PS is also variably present across the larger isolate collection, being absent from an additional 18 strains, so is likely not an essential factor for infection, although it may be required for full virulence.

It remains uncertain why the reported incidence of melioidosis in both Africa and the Americas is far lower than that in other regions where it is endemic, such as Australasia and Thailand. Despite the potential for lower virulence, as shown by the virulence gene profiles, all three cases from Madagascar were blood culture positive and two were fatal (16, 62), suggesting that African *B. pseudomallei* strains possess full virulence. Decreased virulence should not be ruled out; however, other explanations may account for the low reported melioidosis rates in Africa and the Americas, including limited awareness of melioidosis in these regions, poor access to modern diagnostic and laboratory facilities for *B. pseudomallei* detection, a lower likelihood of exposure in the general population because of differences in agricultural practices, or a lower incidence of risk factors in the population (5). A final and noncontradictory factor is that *B. pseudomallei* may indeed be far less common in the environments of Africa and South America than in those of northern Australia and Southeast Asia, although recent modeling suggests that the African and South American environments are permissive for the presence and persistence of *B. pseudomallei* (32); environmental surveillance studies are under way to assess this possibility. Future studies concentrating on functional virulence assessment and genomic analyses of additional *B. pseudomallei* strains from these regions are needed to ascertain the true rate of melioidosis and determine if these strains have maintained their full virulence potential.

### Conclusions.

Despite high levels of recombination in *B. pseudomallei*, sufficient signal exists across the genome to robustly determine the geographic origins of strains. In this study, we used WGS to uncover diverse MLST profiles, ITS types, and variation in the virulence gene content of African isolates. Such diversity strongly supports the premise of long-term endemicity of *B. pseudomallei* in Africa and rules out its recent introduction. Phylogenomic analysis revealed that African *B. pseudomallei* formed a separate clade that was closely related to South American strains, with this clade residing within the deeper Asian clade, suggesting an ancestral Asian origin for *B. pseudomallei* in Africa and the Americas. Phylogenomic analysis also showed that the South American strains emerged more recently within the African clade, an observation supported by the single ITS type (G) of all of the American isolates characterized to date. Anthropogenically driven factors provide a plausible explanation for *B. pseudomallei* transmission to these regions, where *B. pseudomallei* was previously not endemic.

## MATERIALS AND METHODS

### Strain selection.

Two *B. pseudomallei* isolates from Madagascar were obtained from clinical cases occurring in 2012 and 2013 (16), with a third isolate obtained from a French patient in Belgium following a visit to Mahajanga, a city on the northwestern coast of Madagascar (62). The two Burkina Faso isolates were both from children who had not traveled outside Burkina Faso and who presented with localized melioidosis without systemic sepsis. All of the strains used in this study are described in Table 1 (see also Table S1 in the supplemental material). For phylogenomic analysis, we used 144 *B. pseudomallei* isolates from diverse geographic locations encompassing Asia (*n =* 20), Australasia (*n =* 116), Brazil (*n =* 2), Burkina Faso (*n =* 2; this study), Madagascar (*n =* 3; this study), and Ecuador (*n =* 1).

10.1128/mSphere.00089-15.1 Table S1  Complete list of *B. pseudomallei* isolates used in this study. Download Table S1, XLSX file, 0.02 MB. Copyright © 2016 Sarovich et al. 2016 Sarovich et al. This content is distributed under the terms of the Creative Commons Attribution 4.0 International license.

10.1128/mSphere.00089-15.2 Table S2  Virulence gene heat map of all of the *B. pseudomallei* isolates included in this study. Download Table S2, XLSX file, 0.1 MB. Copyright © 2016 Sarovich et al. 2016 Sarovich et al. This content is distributed under the terms of the Creative Commons Attribution 4.0 International license.

10.1128/mSphere.00089-15.3 Table S3  African and South American isolates in the *B. pseudomallei* MLST database and their corresponding allele profiles. Download Table S3, XLSX file, 0.01 MB. Copyright © 2016 Sarovich et al. 2016 Sarovich et al. This content is distributed under the terms of the Creative Commons Attribution 4.0 International license.

### MLST.

MLST of all of the isolates was performed either *in silico* from WGS data by exporting the consensus sequence from the MLST loci with GATK (63) or by Sanger sequencing as previously described (64).

### WGS, variant identification, and phylogenetic reconstruction.

Genomic DNA was extracted from the five African genomes as previously described (65). High-quality DNA was subjected to paired-end WGS with the Illumina HiSeq2000 instrument and NextEra XT library preparation to an average depth of 65× (range, 50× to 80×). Publicly available *B. pseudomallei* genomes were downloaded from NCBI and, where required, converted to simulated Illumina reads with ART (version VanillaIceCream, 85× coverage, paired-end and quality shifts of 10) (66). *B. pseudomallei* MSHR1655, a sequential isolate from a chronic-carriage infection that has undergone genome reduction (61), was excluded from this analysis to maximize core genome size; all of the other available genomes of acceptable quality were included. Core genome, orthologous, biallelic SNPs were identified by GATK v3.1 (63) within the comparative genomics pipeline SPANDx v2.7 (75) by using default parameters. The *B. pseudomallei* K96243 reference genome (67) was used for read mapping by BWA v0.6.2 (68) (GenBank accession numbers BX571965 and BX971966). Maximum-parsimony analyses were performed by a heuristic search with PAUP v4.0b10 (69). Trees were bootstrapped in PAUP using 200 replicates.

### 16S-23S rDNA ITS typing.

The ITS types of the five African isolates were determined from whole-genome sequences. Briefly, genomes were assembled with an in-house assembly pipeline (38) or retrieved from the NCBI genome database. BLAST (70) was used to align the *B. pseudomallei* ITS types (E, C, and G) against the assembled genomes. All of the alignments were manually inspected to determine the ITS types. Where BLAST results were ambiguous (because multiple ITS types were present in a single genome), short-read data were aligned against ITS types E, C, and G using SPANDx and alignments were visually inspected with Tablet v1.15.08.25 (71) to determine the ITS type(s) and their allelic abundance.

### Virulence gene and accessory gene screening.

To assess the virulence potential of the five African strains, we developed an *in silico* virulence gene panel comprising all known *B. pseudomallei* virulence determinants (see reference 52 for a review). All of the genes in this panel are described in Table S2 in the supplemental material. The tBLASTn (72) algorithm was used to assess amino acid identity. BLAST matches were normalized by calculating the BSR (73) with *B. pseudomallei* K96243 as the control. A standard BSR cutoff of 0.70 was applied to call a virulence factor present; loci with scores below this cutoff were considered absent or highly variable. BRIG v0.95 (74) was used to visualize synteny and gene presence or absence across the genome.

### Data availability.

The whole-genome short-read data generated in this study are available in the NCBI Sequence Read Archive under accession no. SRP067905. All of the ST profiles have been submitted to and are available in the *B. pseudomallei* MLST database (http://pubmlst.org/bpseudomallei).
